# SXRF for Studying the Distribution of Trace Metals in the Pancreas and Liver

**DOI:** 10.3390/antiox12040846

**Published:** 2023-04-01

**Authors:** Marko Z. Vatamaniuk, Rong Huang, Zeping Zhao, Xin Gen Lei

**Affiliations:** 1 Animal Science Department, Cornell University, Ithaca, NY 14853, USA; 2 Cornell High Energy Synchrotron Source (CHESS), Cornell University, Ithaca, NY 14850, USA

**Keywords:** transition metals, X-ray fluorescence, pancreas, liver

## Abstract

Transition metals such as iron, copper and zinc are required for the normal functioning of biological tissues, whereas others, such as cadmium, are potentially highly toxic. Any disturbances in homeostasis caused by lack of micronutrients in the diet, pollution or genetic heredity result in malfunction and/or diseases. Here, we used synchrotron X-ray fluorescence, SXRF, microscopy and mice with altered functions of major antioxidant enzymes to show that SXRF may become a powerful tool to study biologically relevant metal balance in the pancreas and liver of mice models with disturbed glucose homeostasis.

## 1. Introduction

About one third of the human proteome contains metal cations in forms of enzymes, cofactors or structural support elements, hence it is not surprising that metal imbalance in cells and tissues strongly correlates with the development of multiple diseases [1]. The essential role of microelements for the normal functional activity of biological organisms is well established [2]. At the same time, prebiotics and probiotics are a very important source of micronutrients [3] and possess neuroprotective effects [4]. The metabolic activities of the body can be impacted by the presence or absence of trace or macro elements [5], and an imbalances in metal homeostasis due to micronutrient deficiencies, environmental pollution, and genetic predispositions strongly correlate with the development of multiple disorders including Wilson and Menkes diseases [6], anemia [7], and neurodegenerative conditions [8,9].

Glucose-stimulated insulin secretion, GSIS, represents one of the central processes in the regulation of the body’s glucose metabolism and homeostasis [10], and its deregulation leads to the development of type 2 diabetes mellitus, T2DM, and metabolic syndrome [11]. Insulin is the major factor in the maintenance of normal blood glucose [11], while insulin signaling and sensitivity in glucose-responsive tissues depends on the physiologically balanced level of Reactive oxygen species (ROS) [12], and thus on the level of the oxidative stress. 

Superoxide dismutases, SODs, are group of metalloenzymes that are essential for protection from oxidative stress. These enzymes require zinc and copper ions for their activity [13]. Glutathione peroxidases, GPXs, are selenium-containing enzymes that degrade hydrogen and lipid peroxides, and act as antioxidants as well [14,15]. Remarkably, the mouse models with knockout or overexpression of selenium-dependent glutathione peroxidase 1 (Gpx1) or copper–zinc-dependent superoxide dismutase 1 (Sod1) experience disturbed glucose homeostasis [16,17,18].

Previously we showed that GPX1 transgenic-overexpressing (OE) mice develop hyperglycemia, hyperinsulinemia, insulin resistance and obesity [17]. The primary reason for this phenotype is attributed to the unexplainable *Gpx1* overexpression in the pancreas (about 20 times), as compared to other tissues, and is associated with the increased function of insulin synthesis and secretion machineries consequently resulting in hyperinsulinemia [19]. Taking into the consideration that each GPX1 molecule contains one atom of Se per subunit [20], a distinct correlation between an islet’s Se level and T2DM-like phenotype becomes evident. A single knockout of Cu, Zn-superoxide dismutase, *Sod1*^−/−^, Se-dependent, *Gpx1*^−/−^, and their double-knockout mouse models have elevated endogenously derived superoxide and hydroperoxide levels, which exert distinct impacts on the body’s glucose homeostasis [16]. Whereas the three knockouts displayed decreased plasma insulin concentrations and islet β-cells’ mass, only *Sod1*^−/−^ showed reduced body weight, increased blood glucose, and blocked GSIS. Given that SODs and GPXs are metalloenzymes, we hypothesize that the observed phenotypes are also associated with altered distribution of Cu, Zn and Se, which, in turn, have an impact on the distribution of other cellular elements in pancreas and liver, thus putatively influencing insulin and glucose homeostasis. Selenium is an important Gpx1 cofactor, and its deficiency results in a decrease in the Gpx1 protein level as well as its activity [21]. Diet supplementation of selenium causes a 20-fold increase in Gpx1 translational efficiency [22], while selenium deficiency reduces Gpx1 mRNA abundance by 20 times [23]. Despite dietary selenium deficiency partially rescuing the OE phenotype, hyperinsulinemia was not eliminated by diet restriction in younger (1–3 months) mice [24], but was partially eliminated in older (5 months old) mice [25]. Moreover, selenium may be involved in complex regulation of upstream targets of insulin homeostasis; the OE phenotype was associated with the increased acetylation of H3 and H4 histones at the proximal Pdx1 promoter [19], suggesting an indirect role of Se in epigenetic regulation of gene expression in pancreatic islets. Thus, disturbed glucose metabolism-related *Sod1^−/−^, Gpx1^−/−^* and OE phenotypes link antioxidant defense mechanisms with glucose homeostasis, and eventually with micronutrients.

Investigations into trace metal concentrations, their spatial distribution and the effect of metal imbalance on the metallome (a “total” metal composition and spatial distribution) in insulin-producing and insulin-responsive tissues will significantly advance our understanding of the role of these metals in insulin homeostasis. Trace metal’s concentration in insulin-producing and insulin-responsive tissues would be very significant in understanding their role in normal homeostasis, and the visualization of trace metal’s spatial distribution in pancreas and liver will expand our understanding of the role of these metals in insulin homeostasis, and unveil the still largely unexplored area of the interrelationships between glucose metabolism and metal homeostasis.

## 2. Materials and Methods

### 2.1. Mouse Genotypes and Diet

Gpx1-overexpressing and knockout mice were originally provided by Y.S. Ho [26], and shared on the same genetic background (129/SVJ × C57BL/6). All experimental mice (males 3–4 months of age) were bred in a Cornell animal facility (Ithaca, NY, USA), fed on a Teklad LM-485 mouse/rat diet, and allowed free access to food and distilled water. Mice were housed in shoebox cages in a constant temperature (22 °C) animal room with a 12 h light/dark cycle. Mice were euthanized with CO2, and then the liver and pancreas were collected and placed on ice before cryofixation. Freshly isolated pancreases were briefly stored on ice before being placed in a Synchrotron X-ray fluorescence (SXRF) frame for scanning. All experiments were approved by the Animal Care and Use Committee at Cornell University (protocol 2007–0008, version 19, approved on 22 May 2020), and conducted in accordance with National Institute of Health guidelines for animal care.

### 2.2. Sample Preparation

In order to find out and to compare spatial distribution of metals in the pancreases and livers of mice without major antioxidant enzymes, we used the following approaches: first, we tested the paraffin-embedded tissue samples, largely used for immunohistochemical visualizations, for SXRF experiments. The possibility of using paraffin-embedded tissues for the detection and spatial distribution of trace elements in different samples opens broad perspectives for any fixed sample, including human biopsy samples; secondly, we prepared samples by cryofixation, using a plunge freezing of samples in liquid propane surrounded by liquid nitrogen [27]. This technique is widespread in electron microscopy, and enables us to preserve an intact internal architecture due to the formation of amorphous ice and the prevention of water crystal formation. These samples with preserved tissue structures are ideal for the intended 3D scanning with advanced resolution. Lastly, we used a special setup which enabled us to scan freshly isolated tissues. Freshly isolated tissue was placed in the wet chamber made between two layers of metal-free Kapton™ film and mounted onto 35 mm slide mounts.

### 2.3. Synchrotron X-ray Fluorescence (SXRF) Microscopy

Samples were placed on a sticky side of Kapton tape. Freshly prepared hydrated tissues were placed in a wet chamber between two layers of Kapton tape. The spatial distribution of micronutrients in all samples was imaged via SXRF at the F3 station at Cornell High Energy Synchrotron Source (CHESS, Ithaca, NY, USA). The 2D microelements’ raster maps were acquired at a 20-µm resolution, a 0.25 s/pixel dwell time using a focused, monochromatic incident X-ray beam at 12.2 keV, and a photon flux of approximately 1 × 10^9^ photons/s. These settings did not cause damage to pancreas within the 2 h scans required for analysis of the full set of genotypes. Element-specific X-ray fluorescence was detected using a Vortex ME-4 Silicon Drift detector (Hitachi, Chiyoda, Tokyo, Japan). Quantifications were carried out using thin film standard data, collected during each experiment, and expressed as µg/cm^−2^. Data were processed with the software Praxes, which was developed at CHESS (Ithaca, NY, USA) and uses PyMCA libraries in batch mode [28].

## 3. Results

### 3.1. Selenium in Pancreas and Liver of Gpx1^−/−^ and Gpx1-Overexpressing Mouse Models

**Figure 1:**
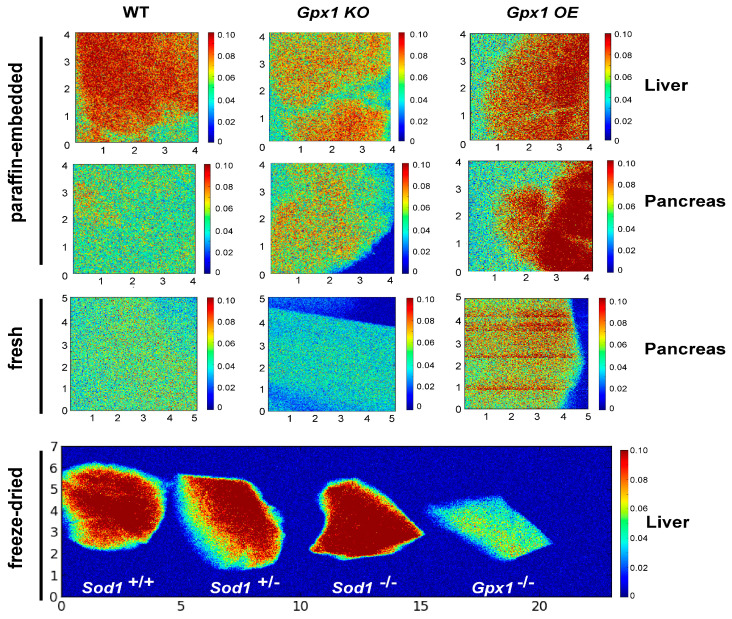
Synchrotron X-ray fluorescence (SXRF) Se imaging in pancreases and livers of wild-type, *Sod1^−/−^ Gpx1^−/−^* and OE mice. The first two rows represent paraffin-embedded samples; the third row represents samples from freshly isolated pancreases. The fourth row represents Se distribution in liver of wild-type and *Sod1^+/−^* (1st and 2nd images from the left, respectively), *Sod1^−/−^* and *Gpx1^−/−^* samples (2nd and 1st image from the right, respectively). The Se map was acquired at 20 µm resolution, with a 0.25 s/pixel dwell time using a focused, monochromatic incident X-ray beam of 12.2 keV. SXRF data were processed with Praxes, a CHESS-developed software for image analysis.

We detected and compared the level of Se in pancreas and liver of wild-type, *Gpx1*^−/−^ and OE mice (Figure 1) in paraffin-embedded tissue samples, samples prepared by cryofixation, and freshly isolated tissues. Despite different sample preparation procedures, the data were comparable, pointing on the various Se distributions in different genotypes.

### 3.2. Micronutrients Visualization in Pancreases of Antioxidant Enzyme-Altered Mice Models

**Figure 2:**
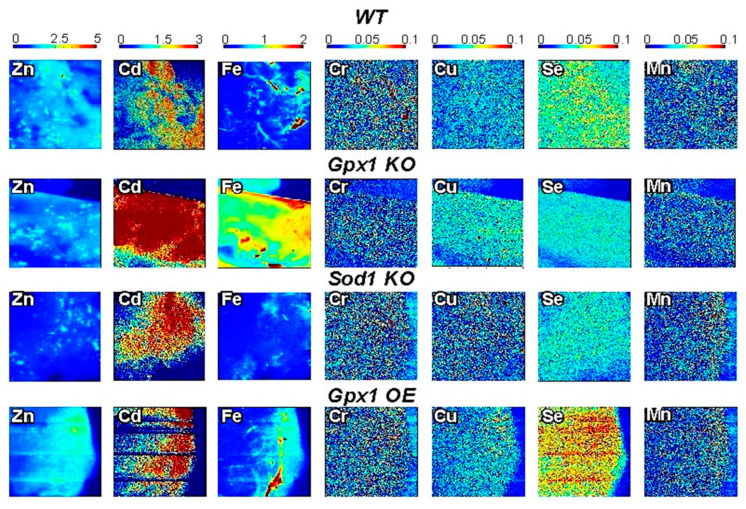
SXRF visualization of seven micronutrients in pancreatic tissue related to the mechanisms of insulin secretion. The redistribution of metals in freshly isolated pancreatic tissue in mouse models overexpressing or lacking major antioxidant enzymes Gpx1 and Sod1. Metal maps was acquired at a 20 µm resolution, and a 0.25 s/pixel dwell time using a focused, monochromatic incident X-ray beam of 12.2 keV. XRF data were processed with Praxes, a CHESS-developed software for image analysis.

Additionally, we distinguished the distribution of seven biologically relevant trace elements in tissue samples of wild-type and genetically modified mice (Figure 2). We detected the distribution of Zn, Se, Cu, Fe, Mn, Cr, and Cd in pancreatic and liver samples of mice models with deleted or overexpressed antioxidant enzymes. Our data show that SXRF is a powerful tool for visualizing differences and analyses of the role of mineral elements in disorders of glucose metabolism. Furthermore, the ability to image the spatial distribution of low abundance elements such as Cu suggests, that the sample preparation procedures can be used for studies of metal homeostasis including metal-promoted/related diseases.

## 4. Discussion

The majority of data of the involvement of trace elements in T2DM were obtained by estimating their concentration in plasma, urine and scalp hair [29,30] using different analytical procedures: atomic absorption spectrophotometry, AAS, anodic stripping voltammetry, ASV [31] inductively coupled plasma mass spectrometry, ICP-MS [32], as well as in situ detection by histochemical techniques, autometallography, radioisotopes and their derivative techniques, magnetic resonance imaging (MRI), X-ray fluorescence [1], and fluorescent sensors [33]. We note that while other methods exist for imaging subcellular details (e.g., electron-probe X-ray microanalysis, electron energy loss spectroscopy, proton-induced X-ray emission), synchrotron X-ray fluorescence, or SXRF-based techniques have the unique advantage of combining great elemental sensitivity due to the absence of a bremsstrahlung background with the ability to image comparatively thick tissue sections at very high spatial resolution [34,35,36,37], and allows visualization of the distribution of many essential cellular metals at the same time point, with high sensitivity in situ. Recently, De Samber and colleagues performed the first SXRF measurements on single, cryofrozen whole islets of Langerhans [38], and the possibility of exploring distribution and content of elements in mouse pancreatic beta-cells was demonstrated as well [39].

In addition to the demonstration of the applicability of sample preparation procedures for SXRF-based studies of metal homeostasis, our data show an important link between the selenium status of the organism and glucose metabolism. Selenium is involved in insulin biosynthesis and secretion by the Gpx1 cofactor, while over-increased Gpx1 activity plays a negative role in the regulation of insulin secretion [19]. In addition, insulin secretion and signaling depend on a cellular redox state [40,41,42], thus linking selenium and Gpx1 with glucose and insulin homeostasis. Interestingly, in humans, high serum selenium and selenoprotein P levels are associated with diabetes biomarkers [43], which is in concordance with animal data [24,44]. We also were able to detect seven micronutrients in freshly isolated pancreases of our mouse models with disturbed antioxidants status, which might be implemented in the development of these phenotypes [16,17,19].

Zinc is involved in normal insulin synthesis and secretion, and thus in maintaining a normal blood glucose level, and its homeostasis is altered in diabetes [45]. Zn is an essential micronutrient that is required for growth and development of all organisms, but is also toxic when it accumulates in cells in excess due to its ability to promote oxidative stress. Interestingly, Zn depletion [46,47], as well as Zn overload [48], may induce and promote oxidative stress-induced apoptosis in islets’ β-cells, pointing to the necessity of balanced concentration of this ion for normal cell function. Thus, Zn also links antioxidant and pancreatic islets biology fields. Manganese is another trace element important for antioxidant defense, and Mn-Sod is the key mitochondrial enzyme of antioxidant defense in a cell. Elevated mitochondrial ROS are involved in muscle insulin resistance [49], while inadequate Mn intake may favor insulin resistance as well [50].

The role of cadmium, iron and chromium in the regulation of insulin homeostasis is not that notable and/or still needs to be explored. Recent reports showed that cadmium exposure decreases fasting blood glucose levels and exacerbates type 2 diabetes in mice [51], while cadmium selectively accumulates in pancreatic islets, thus altering islet function, and, likely contributing to dysglycemia [52]. Iron overload is a risk factor for diabetes. The link between iron and diabetes was first recognized in the pathologic conditions, hereditary hemochromatosis and thalassemia, but high levels of dietary iron also impart diabetes risk [53]. Chromium is an important factor for enhancing insulin activity. Studies show that people with type 2 diabetes have lower blood levels of chromium than those without the disease [54], though newer human studies have reported no effect of chromium supplementation on the attenuation of the risk of diabetes [55].

The understanding of ion distribution and redistribution in normal and in glucose metabolism-disturbed phenotypes will significantly improve our insights about the role of metals in the insulin synthesis and/or release pathways, which are still not completely understood. The mouse models lacking major antioxidant enzymes Gpx1 and Sod1, their double knockout, and Gpx1-transgenic mice will be within our scope to understand the potential involvement of different metals in pancreatic malfunction, and their link with glucose metabolism.

In order to solidify these findings and to find out the underlying mechanisms of deep transcriptome sequencing, RNA-seq of mRNA for genome-wide comparison of gene expression profiles and identification of transcriptional targets of already known transporters will be the next goals to achieve. Through the analysis of RNA-seq data from different glucose metabolism-disturbed phenotypes, the identification of specific gene networks involved in the interrelations between glucose metabolisms and trace metal homeostasis seems a feasible aim.

## 5. Conclusions

Analysis of trace metal’s distribution in the pancreas, its comparison with the expressions of genes involved in trace metal’s homeostasis, and its correlation with the functional abilities of pancreatic islets isolated from different glucose homeostasis-disturbed phenotypes will give answers to many previously unaddressed questions, such as the role of micronutrients in insulin synthesis, secretion, and sensitivity, and will highlight pathways involved in these processes. This novel approach will provide information about the interrelations between trace metal homeostasis, native antioxidant defense, and its importance to glucose metabolism-related diseases and complications. Thus, we hope that these studies will spawn research on transition metal homeostasis related to islets’ β-cell biology, obesity and diabetes.

## Data Availability

Data are contained within article.
